# The effect of animated Sci-Fi characters’ racial presentation on narrative engagement, wishful identification, and physical activity intention among children

**DOI:** 10.1093/joc/jqad030

**Published:** 2023-10-25

**Authors:** Amy Shirong Lu, Melanie C Green, Dar Alon

**Affiliations:** Health Technology Lab, Department of Communication Studies, College of Arts, Media, and Design, Department of Health Sciences, Bouvé College of Health Sciences, Northeastern University, Boston, MA 02115, USA; Department of Communication, University at Buffalo, Buffalo, NY 14260, USA; Harvard T.H. Chan School of Public Health, Harvard University, Boston, MA 02115, USA

**Keywords:** racial ambiguity, social identity, narrative engagement, wishful identification, physical activity

## Abstract

Characters play an integral role in animated narratives, but their visual racial presentation has received limited attention. A diverse group of U.S. children watched a 15-min physical activity-promoting animated Sci-Fi narrative. They were randomly assigned to one of three conditions, which varied the lead characters’ racial presentation: realistic racially unambiguous (Original: White children, Black mother), realistic racially ambiguous (Ambiguous: All with brown skin without specified race/ethnicity), and fantastical racially ambiguous (Fantastical: All with brown skin with fantastical hair-and-eye color schemes). We assessed narrative engagement, wishful identification, and physical activity intention. Controlling for social desirability and multigroup ethnic identity, children who watched Fantastical characters showed significantly higher narrative engagement than those who watched Original characters, but they did not statistically differ from those who watched Ambiguous characters. Structural equation modeling indicated that narrative engagement and wishful identification fully mediated the racial representation effect (Fantastical vs. Original) on physical activity intention.

Animated narratives are pervasive in children’s media. Nine out of the top ten highest-grossing North American children’s movies between 1995 and 2022 were animated (Statista, 2022). Compared to live-action counterparts, the simplified and stylized rendering of the human figure juxtaposed with theatrically exaggerated movements in animation can be developmentally appropriate features for delivering stories to children. Animation’s integration of visual imagery and auditory information is frequently well-received by children (Hayes, 2008).

Media narratives designed for children can promote prosocial goals and positive behaviors (Mares et al., 2008), especially during the middle childhood phase when children become more empathetic (Eisenberg et al., 1987; Mah & Ford-Jones, 2012). Furthermore, the representation of different races in these narratives can affect viewers’ perception and reception (Mastro, 2017), including the extent to which they become engaged in and persuaded by these narratives. Despite the abundance of narrative persuasion research, there has been a paucity of empirical research on the impact of animated characters’ racial presentation on children. Few studies experimentally manipulate characters’ racial presentation or include diverse groups of children as participants. Given the potentially significant effects of media representation, how should entertainment-education designers present characters’ race to appeal to and/or persuade an increasingly diverse mass audience? We attempted to address this question by examining how lead characters’ racial representations in an exercise-motivating Sci-Fi animation influenced children’s narrative engagement, wishful identification, and physical activity intention.

Multiple content analyses have empirically investigated the racial presentation of animated characters in children’s media (e.g., Faherty, 2001; Klein & Shiffman, 2006; Robinson & Anderson, 2006) and identified the crucial issue of the lack of representation of individuals from minoritized racial/ethnic backgrounds. While content analysis is effective to describe media representation on a grander scale, the responses from diverse audiences were not documented. Additionally, researchers seldom experimentally manipulate the characters’ racial presentation due to the technical difficulty of changing a single element (character appearance) in an existing animated program. Finally, most perception studies did not control for demand characteristics (Orne, 2009), which are cues that can bias the validity of research findings when people are potentially aware of a study’s purpose and distort the answers accordingly. For example, when told about a study’s hypothesis, participants tended to respond in ways that confirmed it (Nichols & Maner, 2008).

To respond to the critique about the lack of diversity among human characters, animation creators brought in more characters of color or adopted the so-called “ambiguously brown” trope (Turner, 2013). The former approach involves designing characters of specific racial backgrounds. Examples include Susie Carmichael (Black girl) from *Rugrats* (1991–2004) and Diego Márquez (Latino boy) from *Dora the Explorer* (2000–2019). The latter approach is more subtle and usually produces characters with different skin tone shades but who lack specific phenotypic features of any particular race (Tropedia, 2022). Examples in Western animation include Liz Allan from the TV series *The Spectacular Spider-Man* (2008–2009) and Prince Naveen from the movie *The Princess and the Frog* (2009). Similarly, anime [a distinctive animation genre primarily produced in Japan (Lu, 2008)] also has its own tradition of *mukokuseki* characters (i.e., characters who are stateless or lack any nationality) (Iwabuchi, 2002), which may permit marginalized groups to project their own identities onto the characters (Whaley, 2015). Examples include Yoruichi Shihouin from *Bleach* (2004–2012) and Akio Ohtori from *Revolutionary Girl Utena* (1997). These characters all share some ambiguous phenotypic facial features with different shades of brown skin, making it difficult to identify their exact racial background other than that they are not White. Sometimes these characters also have vibrant colors in their hair and eyes, carrying an unreal and fantastic flair.

In the United States, though, while such a trend of depictions occurred as part of the “browning of America”, its cultural authenticity has been called into question. Scholars warned about the potential for stereotypical depiction under the guise of identifiable presence (Aldama, 2020; Leon-Boys & Valdivia, 2021). Similarly, while ambiguous-race characters were present in many children-oriented STEM education programs, there have been doubts about whether children of color would indeed perceive these characters as their own by projecting themselves onto the characters, or whether they would still perceive these characters as “others” (Aladé et al., 2021). We propose to empirically examine the potential benefits of such presentation in animated narratives, as simple visual adjustments such as ambiguous racial presentations and fantastic coloration may help bring more diversity to animation character design. When used in health-promoting programs, these presentations may have the potential to positively influence the health outcomes of children of color.

We propose that the racial ambiguity manipulation and use of fantastic coloration in character design may help increase diverse audiences’ narrative engagement and wishful identification, thereby increasing the persuasive outcome. We examine 8- to 12-year-old children, who are in middle childhood. This developmental stage features children being more concerned about others out of their genuine empathic feelings (Eisenberg, 1992), but has been understudied in the realm of positive media effects (Mares & Woodard, 2005; Strasburger et al., 2009). This age also signifies a crucial period in the development and formation of racial identity, especially among children of color (Hudley & Irving, 2012). During this period, they move toward racial classification, as well as recognizing social stereotypes associated with racial groups (Quintana, 1998). For children of color, the accumulation of small acts of discrimination and microaggressions may lead them to question the desirability of their own race and negatively impact their development of self-esteem (Swanson et al., 2009). Therefore, seeing animated lead characters of color positively represented on screen may impact identity formation as a potentially beneficial resource in both self and social contexts.

In the domain of physical activity promotion, previous studies on narratives’ behavioral impact mostly targeted the 8- to 12-year age group due to additional developmental concerns. Without health intervention, children with obesity in this age group are highly likely to become obese young adults (Whitaker et al., 1997). Additionally, children younger than eight have limited cognitive capacities in responding to survey questionnaires (Borgers et al., 2000) while children over 12 have entered early adolescence, a period of physical and emotional change that may require different intervention strategies (Centers for Disease Control and Prevention, 2010).

Our study is based on theories from cognitive psychology concerning racial ambiguity, social identity theory, and the common ingroup identity model in the context of narrative persuasion and health communication with a developmental focus. We empirically test our hypotheses via a randomized experiment, employing carefully developed stimuli among a diverse sample of children in middle childhood, with the hope to start a systematic empirical investigation of effective design strategies for animated narrative characters.

## Narrative, animation, and health communication

### Narrative and animation

Narratives have the potential to influence people’s cognition, affect, and behavior. With their unique immersive qualities, such as the potential to mentally transport audiences into a narrative world (Green & Brock, 2000), narratives make health behavior change seem interesting and relevant to the target audience. Narratives can provide characters as role models, facilitate mental simulation of difficult aspects of health outcomes through characters, and reduce the perceived behavioral barriers through reduction in counter-arguments and resistance, resulting in a stronger narrative-aligned attitude, more positive subjective norms, and greater perceived behavioral control over performing healthy behaviors more consistently (Green, 2006; Lu, Baranowski, et al., 2016; Miller-Day & Hecht, 2013; Murphy et al., 2013). Given the strong potential of narratives, it is crucial to ensure that the benefits afforded by narrative-based health communication can be applied across the different racial and developmental spectrums.

Most of the narratives used in previous work are live-action videos or written texts. Animated narratives have received little attention partially due to the lack of animated health behavior-changing narratives, along with the greater costs of animation production in general (e.g., Keller et al., 2022; Winder et al., 2012). Almost all animations used in health communication research with children are short non-narrative educational videos instead of the typical entertainment narratives. For example, such videos are used to reduce children’s anxiety before unpleasant medical procedures (Szeszak et al., 2016). The assumption is that animation presents a lower cognitive load for information processing (Höffler & Leutner, 2007).

Recently, studies have begun examining children’s responses to animated narratives for health behavior change (e.g., exercise). This work has identified various content features that motivate behavior change, including positive role models with supernatural powers and extraordinary actions whose motivation is aligned with exercise (Davies et al., 2016; Lu, Buday, et al., 2016; Lu et al., 2019). When such narratives are coupled with exercise-inducing active video games, which are frequently designed without narratives (Lu & Kharrazi, 2018), children have engaged in significantly greater levels of physical activity (Sousa et al., 2020).

### Narrative engagement and wishful identification as mediators

*Narrative engagement* refers to the level of attention, emotion, or other cognitive resources dedicated by individuals to immerse themselves into a narrative (Busselle & Bilandzic, 2009). Narrative engagement is similar to and highly correlated with narrative transportation (Green & Brock, 2000), but is based on a mental models approach and uses a measurement scale expanded into four subdimensions. These subscales include both cognitive and emotional elements: narrative understanding, attentional focus, emotional engagement, and narrative presence (Busselle & Bilandzic, 2009). The narrative engagement scale can be particularly useful for measurement of visual narratives such as animations and movies. Additionally, in previous work, it had good internal consistency among children and was found to mediate the narrative influence on exercise motivation (Alon et al., 2021), especially when the lead narrative characters’ body shapes were made similar to children. Through narrative engagement, people’s knowledge, attitude, and behaviors can be changed through expanding the range of their imagined possible selves. Positive portrayals of characters of color may help improve the evaluation related to the group those characters represent (Sanders & Banjo, 2022). Therefore, we believe that effective racial presentation of (especially lead) characters would also help produce positive change among children of color.

As part of the social learning process (Bandura, 1977), animated narratives offer children many characters as potential role models. *Wishful identification* refers to the young audience’s desire to become the narrative characters or to mimic their actions (Hoffner, 1996). Wishful identification goes above and beyond the level of similarity-based liking (Cohen, 2001) and reflects children’s desire to be or act like a character and emulate their behavior (Hoffner & Buchanan, 2005). While wishful identification does not necessarily involve perspective-taking as identification does, as part of the vicarious experience, it can exert a powerful influence on young people [e.g., prosociality (Arunrangsiwed et al., 2018; Ramasubramanian & Kornfield, 2012)]. Therefore, when children see characters doing adventurous activities, they are more likely to develop wishful identification toward these characters, whom they may look up to as role models to emulate. This wishful identification may be more likely when children perceive the characters to be part of their ingroup, a perception that may be heightened by visual racial similarity or similarity combined with fantastical features. Consequently, they are more likely to adopt these characters’ beliefs, attitudes, and behaviors as a result of wishful identification.

To sum up, the racial presentation of lead characters as role models can influence both narrative engagement and wishful identification (Bond & Drogos, 2014; Tolbert & Drogos, 2019), which may subsequently influence persuasion outcomes. These two variables have been found to act as mediators of a narrative’s effect on audiences (Bilandzic & Sukalla, 2019; Bond & Drogos, 2014). We believe that similar effects will be observed in this study, especially when the characters look like children of color as opposed to White characters.

## Characters, social identity theory, and common ingroup identity model

According to social cognitive theory (Bandura, 1977), characters can serve as role models, and these role models can be especially influential when young audiences are engaged with and would like to emulate the character. As a major driving force in a narrative, characters serve as an internal source of information or beliefs (Green & Brock, 2000). Positive role models (characters who demonstrate a desired behavior such as engaging in positive health behaviors) can motivate audiences toward beneficial attitude and behavior change (Singhal & Rogers, 2012). These effects have also been demonstrated in children (de Leeuw & van der Laan, 2018).

According to social identity theory (Tajfel & Turner, 2004), part of individuals’ self-esteem derives from their group membership, and individuals are motivated to maintain a positive self-image. Thus, people prefer to see positively depicted ingroup characters (Knobloch-Westerwick & Hastall, 2010). Since individuals from minoritized racial/ethnic backgrounds have been repeatedly portrayed more negatively than White characters (Mastro, 2008; Tukachinsky et al., 2015, 2017), they may have a more difficult time finding positive role models in media and may be less likely to identify with characters who look different from themselves, even when these characters are depicted positively. On the other hand, when they see positive lead characters that somewhat resemble them, they may also be more likely to identify with them.

The common ingroup identity model, an extension of the social identity model through self-categorization theory, provides additional theoretical justification (Gaertner & Dovidio, 2000). It proposes that intergroup bias can be reduced if members of different groups can be made to perceive themselves to be part of the same group through salient features shared by all groups (Gaertner & Dovidio, 2000; Gaertner et al., 1993; Nier et al., 2001). In the context of character design, when animated characters are presented as racially ambiguous and bear salient, though not necessarily distinguishable, phenotypical features of marginalized racial groups, audiences from these groups may be more likely to perceive the characters as part of their ingroup and identify with them more, resulting in a more effective persuasive outcome.

## Race, racial ambiguity, and projection of an individual’s own race

### Racial categorization

Categorizing people into different races based on phenotypical features is still debatable, as race is now understood as a social construct and its categorization varies by culture (Gaither et al., 2022). Nonetheless, people still categorize others based on race unintentionally (Cosmides et al., 2003). The race of another individual is encoded automatically (Fiske & Neuberg, 1990), with the face being the primary visual cue (Bruce, 2017). As the animated character faces may not contain as much detail as live-action characters, these faces are similar to caricatures, displaying a stylized presentation of racially distinctive facial features (Byatt & Rhodes, 1998), subjecting animated characters to audiences’ automatic and sometimes conscious racial categorization.

### Racial ambiguity

Most racial ambiguity studies have been framed in an in- vs. out-group context using real or synthetic adult faces. For example, researchers have used computer software to alter brightness and/or facial features in photos. The results have been inconclusive regarding how people perceive racially ambiguous faces (Young et al., 2020), and memory for these faces depends on the extent to which viewers consider such faces to be among their racial ingroup (Pauker et al., 2009). Racially ambiguous faces are remembered more easily if categorized as ingroup or same-race faces. This process is frequently moderated by the viewer’s own race, with Whites more likely to categorize the ambiguous faces as out-group members while Blacks are more likely to categorize them as ingroup (Gaither et al., 2016). Others consider this a more nuanced and complicated process (Pauker et al., 2018).

Racially ambiguous characters appear to be pervasive. An investigation of primary characters in video games for health has identified almost 30% of the human or human-like characters as racially ambiguous (Lu & Kharrazi, 2018). Another found up to 70% of media characters in food company websites and apps for children to be racially ambiguous (Hurwitz et al., 2019). Together with the aforementioned “ambiguously brown” trope, these suggest that production teams are attempting to appeal to a diverse audience by creating salient phenotypical features in characters that people from a minoritized background may perceive as their ingroup members, according to the perspective of the common ingroup identity model.

Racial ambiguity is not the same as *mixed race*, which is the result of ancestors with multiple different racial backgrounds and thus cannot be altered. Instead, racial ambiguity refers to perceivers’ difficulty in assigning an individual to a certain racial category (though many also find mixed-race people to be ambiguous). Ambiguity can also be artificially created by changing different character visual aspects. While mixed-race faces are perceived to be more attractive (Rhodes et al., 2005), the reception of racial ambiguity is relatively understudied.

Of the few studies investigating people’s perception of racial ambiguity, one found that racially ambiguous synthetic characters are as effective as racially matched ingroup characters in generating identification and positive consumer attitudes (Appiah & Elias, 2011). Another found objectively measured positive responses from audiences toward racially ambiguous models relative to unambiguous ones (Read, 2020). Children received ambiguous faces well when the face shared their own phenotypic features. For example, when a health game character was designed with the facial characteristics of both Black and Latino features, 10- to 12-year-old Black and Latino children had greater game story immersion and better health outcomes than White children (Lu et al., 2012).

The generally positive response toward racially ambiguous faces has several possible explanations. One explanation concerns the opportunity offered to audiences (especially audiences of color) by racially ambiguous characters to mitigate negative emotional responses associated with the lack of the presence of people of color. The common ingroup identity model suggests that these characters may be more likely to be perceived as similar to the audience than characters who are clearly members of outgroups. Therefore, the audience is more likely to have an ingroup association, or at least a more positive association, with these characters (Pauker et al., 2009). We suggest that similar effects may occur with racially ambiguous fantastical characters, who even more clearly are not part of an identifiable outgroup.

Another explanation is closely related to ambiguous faces in animation. Specifically, the Own Race Projection theory argues that, when audiences encounter racially ambiguous animation characters, they are more likely to project their own racial identities onto them because audiences’ own racial background is a stable, accessible trait that guides perceptions (Lu, 2009). Similarly, scholars in comic research have suggested (McCloud, 1993) that the character faces create a type of vacuum that viewers attempt to “fill up” with their own identities. In other words, people may be likely to perceive racially ambiguous or fantastical characters as ingroup members through self-projection and thus are more likely to identify with them.

In practice, since animated characters in media programs are typically not customizable for different populations, it is important to explore the differential effect of the racially ambiguous vs. racially unambiguous presentation of the lead characters. Theoretically, when the characters are created to look racially ambiguous (and all other factors are held equal), we would expect greater engagement with the story, increased wishful identification with the characters, and greater persuasion outcomes, relative to racially unambiguous presentation of the same characters, especially among a sample mostly from minoritized racial/ethnic backgrounds. Thus,

H1: In the context of animation intended to promote physical activity, compared to racially unambiguous lead characters (featuring White children and a Black mother), presenting all as racially ambiguous characters will produce (a) greater narrative engagement, (b) higher wishful identification, and (c) better physical activity intention among children of color.

## Fantastical appearances: character color design

Animation provides additional freedom to present characters in ways that go beyond real-world appearances. The character’s facial features, such as eye and hair color, can be presented using different color palettes, ranging from realistic human colors (e.g., brown eyes, black hair) to fantastic and vibrant colors (e.g., orange eyes, purple hair). Choosing the optimal color palette has become a crucial aspect of character design, which may influence the racial perception of characters and reception of the entire narrative (Elliot et al., 2015). Similar to racially ambiguous characters, characters with fantastical appearances may provide even more opportunity for a wide range of audiences to engage with them, because they have further removed specific racial cues, thus enabling audiences to project their own identities onto the characters more fluently. Audiences typically prefer to identify with a story’s heroes, and from the common ingroup identity model’s perspective, because the characters do not reflect a specific outgroup (e.g., a realistic other racial group), their salient fantastical looks may be more likely treated as ingroup members by a wide range of audiences and especially audiences of color.

Previous formative analyses of children’s narrative preferences suggest that 8- to12-year-old children prefer the Sci-Fi genre and want to see supernatural power and extraordinary actions in a story (Lu et al., 2019). Additionally, fantastical content is already common in 2- to 6-year-old children’s programs (Taggart et al., 2019). Thus, in addition to enabling own-race and ingroup projection, a fantastical color palette can also be congruent with a Sci-Fi story and may therefore increase engagement.

H2: In the context of a Sci-Fi animation to promote physical activity, fantastical racially ambiguous characters will produce (a) greater narrative engagement, (b) more wishful identification, and (c) better physical activity intention compared with racially unambiguous lead characters and realistic racially ambiguous characters among children of color.

Narrative engagement and wishful identification may also mediate the association between the racial presentation of Sci-Fi characters and children’s physical activity intention. Effective lead character racial presentation in design would help children engage more with the Sci-Fi stories and develop more wishful identification with the characters, making them form intentions to imitate their preferred characters as role models (i.e., observational learning). Thus,

H3: The effect of Sci-Fi animated characters’ racial presentation on children’s physical activity intention (e.g., greater intention for racially ambiguous and fantastical than racially unambiguous characters) will be mediated by narrative engagement and wishful identification.

## Methods

### Recruitment

We partnered with eight afterschool activity centers serving underrepresented communities in the Greater Boston area. We disseminated information sheets through the center site coordinators, who provided the parents and children with our screening questionnaire, consent, and assent forms. Children were eligible if they could speak and understand English, were between 8 and 12 years of age, and were able to complete our protocol. The study was authorized by the Northeastern University Institutional Review Board. All participants gave assent, and their parents gave their consent to permit the research team to work with their children.

### Procedures

This study is divided into two phases: Phase 1, the formative study and production phase, took place from February to December in 2018 and Phase 2, the main study data collection phase, took place from January to April in 2019. There was no overlap between Phase One and Phase Two participants.

Both Phase One’s formative study portion and Phase Two took place in the same afterschool centers, all of which served low-income and immigrant families. Almost all participants came from underrepresented backgrounds. The research assistants (RAs) first traveled to each center to measure participants’ height and weight since the project was related to motivating physical activities among children. Body mass index (BMI) was calculated using the standard method [height(m)/weight(kg)^2^], and BMI percentile was calculated using the CDC growth charts (Kuczmarski et al., 2000).

Phase One aimed to enable the children and the research team to work closely with a professional production studio in an iterative fashion to create stimuli for Phase Two. Qualitative data were collected through multiple informal, casual conversations with around 30–40 8- to 12-year-old children. More specifically, the production studio would generate different versions of artwork on a bi-weekly basis. The research team would then print them out and inquire about children’s insight into the perceived racial and ethnic backgrounds of these characters.

The conversation with each child typically lasted between 5 and 10 min before they left for their other afterschool activities. The RAs would show these images to each child individually and ask their thoughts about the character’s race and ethnicity. As the child commented on the characters, the RAs would jot down the gist of their comments and the perceived racial and ethnic background of the characters. Between five and seven children provided their insight each week on average. Once the information from children had been collected, the research team would meet separately in the next week to discuss how these opinions could be integrated into the revision of the character design as well as offer additional revision suggestions for the characters. The first author collected all the inputs and communicated these to the production studio through email in the form of verbal as well as visual sketches as part of the instruction.

Based on these inputs, the production studio would then make modifications accordingly and the research team would then repeat the cycle of gathering children’s insight in the next week. This process lasted around 6 months until the final versions of the characters had been pinpointed. The production studio then moved on to the production of the stimuli for the Phase Two main study.

#### Phase One narrative production: challenges and solutions

A professional production studio, FableVision, was scheduled to create a 15-min Sci-Fi animation titled *Ataraxia*. The story is set in a dystopian future in which a traveling merchant woman adopted a twin brother and sister with superpowers and raised them along with her own child. However, an evil ruler kidnaps the twins in hopes of creating an army to expand his kingdom. The child of the adoptive mother (“you”, who is presented as an androgynous shadow to allow children’s self-projection; see Supplementary Figure 3) grows up with the twins and is inspired by the twins’ good deeds as role models. This character, who is intended to help the viewer feel like part of the story, begins developing superpowers through exercising like the twins throughout the story and must learn to control these powers to save their siblings and the world.

Our initial plan was to create a racially unambiguous condition as the original condition (the baseline comparison point). We discussed how to cast the race of the twins vs. the adoptive mother figure and conducted some pilot design studies. There were two options: One was to create White twins and a Black mother, and the other was to create Black or Latino twins with a White mother. The first version cast the twins as White, and almost all participants agreed upon their racial representation upon seeing them. Their rationale centered primarily on the fact that the twins have ginger-colored hair and blue eyes and white skin. We also cast the mother figure as a Black person, whose phenotypic features (e.g., her nose shape was designed to have a broad base) and hairstyle (e.g., dreadlocks) were indeed perceived by most children as being Black during our formative studies as well.

The other option was to swap the racial and ethnic backgrounds between the twins and the mother, to cast the twins as either Black or Latino and the mother as White to form another racially unambiguous condition. While we were wary of the potential perception that this would fall into the so-called “White Savior Trope,” we faced another significant issue of designing racially specific Black and Latino children characters. To our surprise and disappointment, it was very difficult, if not impossible, to create a pair of racially unambiguous Black/Latino children characters whom children from these backgrounds would agree upon about their race and ethnic origins.

We tried to adjust the character design multiple times, but it was difficult to get more than half the children, and even the RAs themselves, to come to agreement with regard to the characters’ specific racial backgrounds, given the level of rendering details from the animated character design. Instead, participants as well as RAs seemed to have a difficult time perceiving the design options as any specific race. Every time we showed the character design to them, we tended to receive more than five to six broad racial categories ranging from Black to Latino to Middle Eastern to Native American to multiracial from these children. We also tried to look into whether these perceptions would correspond to their own racial or ethnic backgrounds. Interestingly, it was difficult to identify any pattern from any specific race group and the race/ethnic identification was more idiosyncratic in that even people from the same background tended to have diverse opinions about the character’s looks.

Therefore, we experienced significant delays in finalizing the characters according to the original production plan. We tried to increase the production budget to be able to find the best design. However, after two extra months of discussion and a huge delay and extra investment in the production company, no consensus could be achieved. Therefore, while we had some success creating a Black mother figure, we were unable to create either racially unambiguous Black or Latino children characters. Given these obstacles, we thought that pursuing this type of design approach could further confound the subsequent Ambiguous and Fantastical conditions and decided to list this as a limitation.

Instead, the production company further tweaked the character designs that evoked the most diverse opinions to form the Ambiguous condition as well as the Fantastical conditions. Once the line shape of the characters had been decided, the production studio also varied different color palettes in these characters, and the RAs, together with children, provided feedback to help determine the best color palette selection. For example, we wanted to ensure that the skin tone was a neutral brown tone for the Ambiguous condition and the Fantastical condition presented colors that were not commonly seen in eyes and hair.

To sum up, the Phase One’s preparatory and formative work aimed to confirm that the characters’ visual features were created appropriately. Our extensive formative interviews with children and discussion among the research group reassured us that: (1) all three versions of the characters were equally attractive (i.e., almost all of the participants agreed that they look friendly, attractive, and interesting); (2) the Originals would look racially unambiguous (i.e., the twins look White and the mother looks Black); (3) the Ambiguous and the Fantastical would both look racially ambiguous (i.e., the children and RA could not form a consensus of what kind of race or ethnicity they belong to and most guessed the characters to be of multiple unknown origins); and (4) the Ambiguous would appear more realistic than Fantastical (which used unrealistic colors for the characters’ eyes and hair) (i.e., the Fantastical characters could be less commonly seen in real life and their color palette made them seem “mysterious” and “unfamiliar”).

The production company then created three versions of the animation that were identical except for the three different racial presentations of the characters. The different racial appearances were accomplished using multiple layers of animation panels to allow only the characters to be modified without changing any other narrative elements. One animation designer created all three versions of the characters to ensure stylistic uniformity: realistic racially unambiguous (Original: White twins and Black mother), realistic racially ambiguous (Both twins and mother were Ambiguous), and fantastical racially ambiguous (Both twins and mother were Fantastical). (see Supplementary Figure 1 for illustration.) Everything else was identical across the three animation versions.

Phase Two focused on the quantitative data collection from children of the same age group, who were randomly assigned to one of three experimental conditions: (A) Original (Racially unambiguous: White children; Black mother); (B) Ambiguous (Realistic racially ambiguous); and (C) Fantastical (Fantastical racially ambiguous). (see Supplementary Figure 1 for A–C character designs.)

As Phase Two participants, children were invited to a classroom where they usually watched TV during afterschool hours. Depending on different afterschool sizes and specific schedules, 4–15 children entered the classrooms in racially mixed groups. The RA team accompanying the participants also came from diverse racial backgrounds. The RAs told the children that they would be watching an animated series and then answering questions about their impression of the animation. To ensure that children watched the narrative without distraction, the RAs asked the children not to talk during the animation program.

After watching the animation, children were given paper copies of questionnaires including demographic information (age, sex, race), Narrative Engagement (NE), Wishful Identification (WI), Physical Activity Intention (PAI), Social Desirability (SD), and the Multigroup Ethnic Identity Measures (MEIM). To minimize the potential co-viewing effect in interracial settings (Banjo et al., 2015), RAs were assigned to match the majority of children’s racial backgrounds in each setting and remained neutral and stayed aside while operating the TVs. After they had completed the questionnaires, each participant received a $25 gift card. RAs flagged participants who simply answered all the questions without reading them (e.g., selecting “1” or “5” for all questions) or who finished excessively quickly (e.g., completing more than 50 questions in less than 3 min), suggesting potential lack of validity (Dominik Johannes, 2019). The flagged questionnaires were examined and later removed from the data analysis.

### Questionnaires

All questions employed a Likert-style response scale (1 = completely disagree, 3 = neutral, and 5 = completely agree). The detailed scales can be found in Supplementary Table 1.

#### Narrative engagement

The 13-item Narrative Engagement scale was adapted for children from the original scale for adults (Busselle & Bilandzic, 2009). A sample item is, “During the story, my body was in the room, but my mind was in the story world” (Cronbach’s α = 0.70). We used a measure validated in previous research that made minor wording changes to ensure that children in this age group fully understood the questions (Alon et al., 2021).

#### Wishful identification

The 3-item Wishful Identification scale was used verbatim because the original scale was developed for children (Hoffner & Buchanan, 2005). Children were asked to think about their favorite character in the story and answer questions. Most children (96%) chose either one of the twins or the mother as their favorite character. Sample questions included “I wish I could be more like him/her” (Cronbach’s α = 0.72).

#### Physical activity intention

The 5-item Physical Activity Intention scale was adopted from a validated questionnaire measuring children’s motivation for undertaking exercise (Alon et al., 2021). Items included: (If there is an active video game with this story as its plot, what would you do?) “I intend to exercise through this active video game” (Cronbach’s α = 0.84).

#### Social desirability

The 9-item Social Desirability scale controlled potential demand characteristics and response bias for participants’ self-reports (Reynolds & Paget, 1983). A sample item is: “I tell the truth every single time” (Cronbach’s α = 0.75).

#### Multigroup Ethnic Identity Measures

The 13-item Multigroup Ethnic Identity Measures scale assessed participants’ degree of identification with their own racial and ethnic group (Phinney, 1992) as another control. Sample items are: “I feel a strong attachment towards my own ethnic group” and “I feel good about my ethnic background” (Cronbach’s α = 0.85).

Due to the extensive formative work in Phase One, we decided not to perform a manipulation check to ensure that children perceived the characters differently, because we did not want to distract from children’s narrative experience (O’Keefe, 2006).

## Results

All participants watched the animation program attentively, and no conversation occurred during or after the show, except some children asked clarification questions while answering the questionnaires. Of the 86 participants who completed the study, we removed 13 children’s responses because their answers were flagged for lacking validity, leaving 73 participants for analysis. We conducted a *post hoc* power analysis using G*Power (Faul et al., 2009) and a similar study published previously (Alon et al., 2021) to determine whether our final N would be sufficient to detect a significant difference between the three conditions for a large effect size (partial η^2^ = 0.14), and found that our sample size would provide a power of 90% (at α = 0.05).

Participants’ average age was 10.0 years (range = 8–12, standard deviation = 1.20), with 41 males (56.2%) and 32 females (43.8%). The sample was primarily Black and Latino, with 39 participants (53.4%) self-identifying as Latino, 21 (28.8%) as Black, 6 (8.2%) as mixed race, 5 (6.8%) who self-identified as “Other”, and one (1.4%) Asian child. Chi-square analysis revealed no statistically significant differences between the three experimental groups (Table 1; *p* values all >.05) regarding their age, sex, racial composition, body composition, Social Desirability, or Multigroup Ethnic Identity Measures.

**Table 1.** Characteristics of the sample. Data expressed as mean (±*SD*) or as frequencies and percentages (*N*=73)

Variables	Original: Racially unambiguous (*n* = 24)	Ambiguous: Realistic racially ambiguous (*n* = 23)	Fantastical: Fantastical racially ambiguous (*n* = 26)	*p*
Age (years)	10.3 ± 1.3	10.2 ± 1.4	9.7 ± 0.9	.12
Girls/boys	14/10	12/11	15/11	.90
Body weight (kg)	39.5 ± 13.2	42.5 ± 12.5	39.9 ± 12.0	.67
Height (cm)	142.4 ± 10.0	144.5 ± 9.6	141.3 ± 9.8	.52
BMI (km/m^2^)	19.1 ± 4.3	20.1 ± 4.6	19.7 ± 4.5	.75
BMI percentile	62.0 ± 30.0	68.0 ± 30.9	70.0 ± 32.0	.64
Social desirability	3.5 ± 0.9	3.6 ± 0.5	3.8 ± 0.7	.38
Race identification	3.5 ± 0.8	3.9 ± 0.6	3.8 ± 0.7	.08
Race, *n*^1^ (%)				.30
Asian	0 (0)	0 (0)	1 (3.8)	
Black	4 (16.7)	10 (43.5)	7 (26.9)	
Latino	15 (62.5)	10 (43.5)	14 (53.8)	
Mixed	3 (12.5)	0 (0)	3 (11.5)	
Native American	1 (4.2)	0 (0)	0 (0)	
White	0 (0)	0 (0)	0 (0)	
Other	1 (4.2)	3 (13.0)	1 (3.8)	

BMI = body mass index.

1 100% for each condition.

Given satisfactory Cronbach’s α values, the items on each scale were averaged. We compared linear data between groups using MANOVA together with (Bonferroni) *post hoc* comparison when needed. H1 stated that racially ambiguous characters (both Fantastical and the Ambiguous characters) should produce higher (a) Narrative Engagement, (b) Wishful Identification, and (c) Physical Activity Intention than racially unambiguous characters (Original), and in H2, we predicted that Fantastical characters would produce higher (a) Narrative Engagement, (b) Wishful Identification, and (c) Physical Activity Intention than either of the other two conditions. The Fantastical group had a statistically significantly greater Narrative Engagement [*F*_(2, 70)_ = 5.25, *p *=* *.01, η^2^ = 0.13], as well as a non-significant (borderline) but slightly greater Wishful Identification [*F*_(2, 70)_ = 2.66, *p *=* *.08, η^2^ = 0.07] and Physical Activity Intention [*F*_(2, 70)_ = 2.65, *p *=* *.08, η^2^ = 0.07] (see Table 2 and Supplementary Figure 2). The other two groups’ values for Narrative Engagement, Wishful Identification, and Physical Activity Intention were slightly or moderately lower. We found that H1(a) was partially supported as only the Fantastical group had significantly greater Narrative Engagement scores than the Original group in *post hoc* comparison of the means with Bonferroni adjustment (*p* = .006), while the Ambiguous group did not (*p* = .585). Our results partially supported H2(a) because the Fantastical Group had greater Narrative Engagement scores than the Original group but not significantly greater than the Ambiguous group. We did not find support for H1(b), H2(b), H1(c), or H2(c).

**Table 2.** Descriptive statistics and one-way multiple analyses of (co-)variance of Narrative Engagement, Wishful Identification, Physical Activity Intention, Social Desirability, and Multigroup Ethnic Identity Measure (MEIM)

Variables	Original: Racially unambiguous (*n* = 24)		Ambiguous: Realistic racially ambiguous (*n* = 23)		Fantastical: Fantastical racially ambiguous (*n* = 26)		*F* (adjusted+)	*p* (adjusted+)	η^2^ (adjusted+)
	Mean	*SD*	Mean	*SD*	Mean	*SD*			
Narrative Engagement	3.00	0.82	3.26	0.61	3.61	0.72	5.25 (3.77)	.01* (.03*)	0.13 (0.10)
Wishful Identification	3.14	1.40	3.52	1.42	3.94	1.37	2.66 (1.38)	.08^#^ (.26)	0.07 (0.04)
Physical Activity Intention	3.39	1.42	3.90	0.94	4.10	0.92	2.65 (1.24)	.08^#^ (.30)	0.07 (0.04)
Social Desirability	3.49	0.92	3.58	0.46	3.77	0.70	0.98	.38 [*p*s < .001*]	0.03
MEIM	3.49	0.80	3.91	0.57	3.84	0.68	2.59	.08 [*p* > .18]	0.07

(Adjusted + values): adjusted significance test values for the first three dependent variables (Narrative Engagement, Wishful Identification, Physical Activity Intention) when controlling for covariates (Social Desirability and MEIM) from MANCOVA.

[*p*s]: significance tests of Social Desirability and MEIM on each of the dependent variables, respectively, from MANCOVA.

* *p* < .05;

# *p* < .10.

We also applied additional MANCOVA models controlling for Social Desirability and Multigroup Ethnic Identity Measures to the main outcomes (e.g., Narrative Engagement, Wishful Identification, and Physical Activity Intention). After we controlled for Social Desirability and Multigroup Ethnic Identity Measures, we still found a statistically significant group effect [*F*_(2, 69)_=3.77, *p *=* *.03, η^2^=0.10] for Narrative Engagement; however, the borderline effects for both Wishful Identification and Physical Activity Intention disappeared (Table 2). Our *post hoc* analyses of the means with Bonferroni adjustment indicated that the Fantastical group had higher Narrative Engagement scores than the Original, but not the Ambiguous groups; we found a similar trend for Wishful Identification and Physical Activity Intention, but the differences between the groups were not statistically significant. Social desirability (after we included it in our model as a covariate) had a statistically significant positive effect (all *p* values <.001) on Narrative Engagement, Wishful Identification, and Physical Activity Intention while Multigroup Ethnic Identity Measure did not (*p*s >.18).

**Figure 1.**
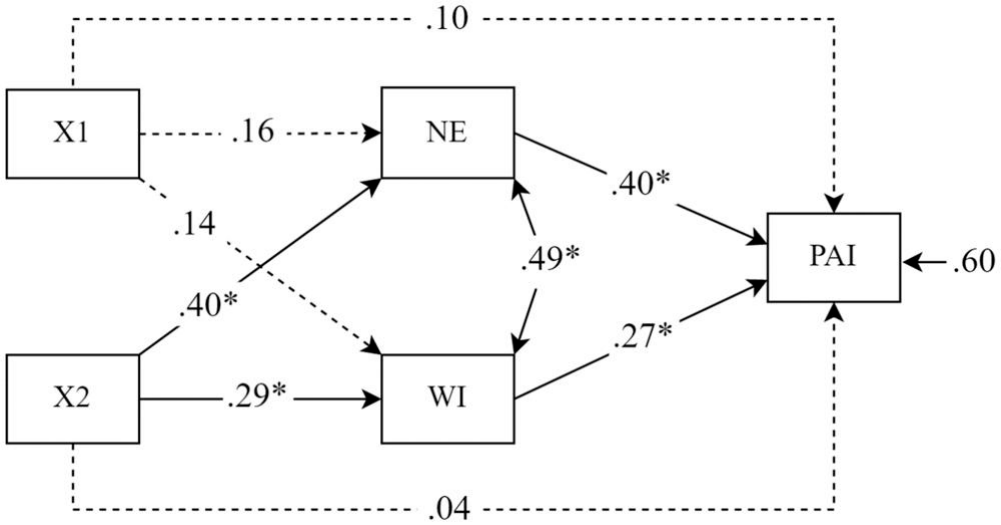
Standardized parameter estimates for the mediation model. *p < .05. The dotted lines represent the non-significant paths. X1: B: Ambiguous vs. A: Original (dummy code). X2: C: Fantastical vs. A: Original (dummy code). NE = Narrative Engagement; WI = Wishful Identification; PAI = Physical Activity Intention.

H3 predicted that Narrative Engagement and Wishful Identification would mediate the effect of the character’s racial presentation on Physical Activity Intention. To accommodate multiple independent and mediator variables, we used structural equation modeling in our mediation analysis to test for associations between the character’s racial presentation and the participants’ Narrative Engagement, Wishful Identification, and Physical Activity Intention. Figure 1 shows the analysis testing the mediation effects of Narrative Engagement and Wishful Identification on the relationship between Racial Presentation (independent variable) and the Physical Activity Intention (dependent variable). (For simplicity, Figure 1 does not show either the indirect effect: X1, X2 → Narrative Engagement, Wishful Identification → Physical Activity Intention or the total effect: X1, X2 → Physical Activity Intention.)

The model analysis was conducted in Mplus V.8.7 (Muthén & Muthén, 1998–2021). This model determines whether the association between the Racial Presentation (independent variable recoded into two dummy codes: X1 = B: Ambiguous vs. A: Original; X2 = C: Fantastical vs. A: Original) and Physical Activity Intention (dependent variable) was mediated by Narrative Engagement and Wishful Identification (mediation variables). To compare all three Racial Presentation categories, a second model was conducted using the same number of parameters with the following two dummy codes to use the Fantastical group as a reference group to highlight the contrast between C: Fantastical condition and the other two: X1 = B: Ambiguous vs. C: Fantastical; X2 = A: Original vs. C: Fantastical. Given our relatively small sample size, we used the Bayesian estimation of model fit to decrease the potential bias in the parameter estimates and indirect effects (Miočević et al., 2018). There were no missing data.

We obtained a good fit for our models [the posterior predictive *p*-value (PPP) = 0.47; CFI = 0.99 and TLI = 0.96]. The model explained 40% of the variance in Physical Activity Intention. Our results suggest that Narrative Engagement and Wishful Identification acted as mediators [for X2 (Fantastical vs. Original) only] for the relationship between Racial Presentation and Physical Activity Intention. While the total effect (c) of Racial Presentation (Fantastical vs. Original) was significant (β = 0.28, *p* = .03), the direct effect of Racial Presentation (Fantastical vs. Original) on Physical Activity Intention (Path c′) was no longer significant (β = 0.04, *p* = .70). However, the indirect effects were significant, indicating full mediation via Narrative Engagement (β = 0.15, *p* = .004) and Wishful Identification (β = 0.07, *p* =.05). We checked the robustness of our overall model and tested the possible interactions of the Social Desirability and the Multigroup Ethnic Identity Measures with the condition variables. These interaction coefficients were not significant, and overall results remained similar. Thus, we obtained partial support for H3 as we found that significant mediation only occurred for the Fantastical group compared to the Original group. Finally, the same model was rerun with the second set of dummy codes that used the Fantastical group as the reference group. Results replicated the previous findings and there were no significant differences between Ambiguous and Fantastical groups on Wishful Identification and Physical Activity Intention in terms of mediation.

## Discussion

This article provides the first empirical insights into the effects of the racial presentation of Sci-Fi animated narrative characters on children’s narrative engagement, wishful identification, and physical activity intention while controlling for social desirability and racial identity. We demonstrated that a character design involving racial ambiguity and fantastic coloration in a Sci-Fi animation helped increase children’s narrative engagement when compared to the character design with racially unambiguous characters (White children; Black mother). Racially ambiguous characters did not show significant increases above the racially unambiguous condition (although they were also not significantly different from the fantastical condition). Similarly, indirect effects emerged such that the greater narrative engagement and wishful identification with the fantastical character improved the persuasive outcome.

This work provides an important contribution to media effects theories by suggesting that fictionalized characters, whose appearance does not reflect a real racial group, can be especially engaging to diverse audiences compared to white-presenting characters, suggesting that such characters may potentially evoke a common ingroup identity. However, we note that we did not directly measure perceptions of ingroup or outgroup status; because we were unable to assess children’s actual thoughts about their perception of the characters, it is possible that these effects might be driven by other factors (e.g., the Fantastical group’s coloring of hair and eyes may have been more appealing, given that there was no Fantastical version of the racially unambiguous characters).

Our data on participant responses to somewhat subtle adjustments to a character’s hair and eye color indicated that the fantastical racially ambiguous condition produced significantly higher narrative engagement than the original racially unambiguous design. This finding is aligned with past observations in animation and game studies (Lu, 2009) that children may relate to character racial ambiguity and fantastical features in the appropriate context. Like the previous studies (Lu, 2009) that examined racial categorization of animated characters, our participants may have projected their own racial identity onto characters of ambiguous race with fantastical coloring, making the characters potentially ingroup members as opposed to the original condition, when the twins were cast as out-group characters. This effect did not emerge strongly with the racially ambiguous condition, perhaps supporting recent critiques of this approach.

We also demonstrated that narrative engagement and wishful identification mediate the effect of Sci-Fi characters’ racial presentation on children’s physical activity intentions, especially when the Sci-Fi characters are fantastical and racially ambiguous when compared to the original racially unambiguous condition. Both variables demonstrate animated narratives’ potential to bring about health changes among children. Although narrative engagement has been shown to act as a mediator previously in studies of animation effects on physical activity (Alon et al., 2021; Sousa et al., 2020), this is the first time its mediation effect has been demonstrated within the context of the racial presentation of an animated character. Likewise, wishful identification mediated the effect between the character’s racial presentation and intention to perform healthy behavior. Both narrative engagement and wishful identification may be crucial factors for future physical activity interventions. Additionally, we controlled for both children’s social desirability and their own racial identification. Future studies should consider including these variables when working with children for rigorous effect exploration.

From a practical perspective, our study also offered some insight into the design and reception of health communication agents in stories for children. Given the higher narrative engagement when characters look fantastical and racially ambiguous when compared to the original racially unambiguous condition featuring out-group children characters, creators should be cognizant of how to present the characters’ race when creating health communication media content for children of color. When the characters’ racial presentation is designed effectively, children of diverse backgrounds may relate to the story and the message better. Creating inclusive character looks could increase children’s narrative engagement and wishful identification and motivate them to engage in exercise and ultimately enhance their health and well-being.

Additionally, in animation, characters often appear in a setting that has its own visual style and color scheme. An engaging animated narrative should ideally have all of its visual elements congruent in nature, thereby increasing the viewer’s processing fluency (Oliva & Torralba, 2007). Thus, a character with unusual hair or eye color may be congruent with a narrative set in another world (such as in science fiction or fantasy stories). Studies suggest that congruency produces more positive persuasive outcomes (e.g., Noseworthy et al., 2011). Although we did not test contextual congruency directly (i.e., we did not include an intentionally incongruent condition), this result was also consistent with the idea of contextual congruency in the character’s color palette. Both the ability to identify with the character and contextual congruency may affect narrative engagement.

Children did not seem as engaged in the narrative when the characters were presented as racially unambiguous when compared to the Fantastical condition. While this Original condition did present multiple characters of different racial backgrounds, the fact that the twins were designed as White characters and only the mother character was Black may have also influenced how this predominantly Black and Latino sample engaged with the narrative. While there was a Black lead character in the narrative, the character is an adult and mother figure to the twins. Children may not respond to an older adult the same way they respond to characters their age. Therefore, they may have treated the White twin characters as out-group members and thus reduced their narrative engagement and wishful identification and persuasive outcome. Another explanation is that they might have seen the White characters simply as less exciting and more ordinary, given the prevalence of White characters on TV. To differentiate these possibilities, future studies could directly ask children about their perceptions of the characters as ingroup or outgroup members.

Additionally, we did not test directly whether the children would perceive the fantastical characters as like themselves or just another unrealistic illustration. Post-survey interview questions may help address this issue in future work. In other words, this study did not directly test different theoretical accounts (such as common ingroup identity) but rather used these theories as possible accounts of what might be going on in children’s minds. Future studies could include these additional character design conditions to further explore the difference between the ingroup presentation vs. the ambiguous presentation options tested here.

Another limitation is that we could not include conditions where the twins appear Black or Latino and the mother looks White or when all characters appear Black or Latino (racially specific presentation of ingroup characters) due to the difficulty of finding an agreement that particular character designs unambiguously represented these groups. This challenge was unexpected and may reflect the general complexity of portraying race in animated narratives, but nonetheless, creating characters that represent specific groups remains an important goal. While the time and budget constraints of our project funding, unfortunately, did not allow us to successfully create these animations for the current study, different illustrators or animation studios may be better able to create more racially specific characters for future work.

We also did not include an incongruent condition (e.g., a fantastical character in an otherwise realistic setting) or other narrative genres. The increased narrative engagement we observed here may be limited to the Sci-Fi genre (or similar genres where fantastical hair or eye colors would be plausible) and not applicable to other types of stories. However, animated stories in general often use a range of color palettes for characters, so it is possible that fantastical coloring could be used across a variety of genres, as long as the character appearance was consistent with the artistic style and other elements of the narrative world.

Due to our relatively small sample size, we could not investigate the effect of the character’s racial presentations on children of all races. This small sample also prevented us from investigating gender differences. While our research demonstrates that the fantastical racially ambiguous condition seems to be more optimal for physical activity promotion, we only measured hypothetical physical activity intention via self-report instead of actual physical activity behavior and the superior effect of the Fantastical condition was only observed when compared to the Original condition. Last but not least, we have only investigated a one-time exposure. Prolonged interaction with media narratives may have different effects.

Despite the limitations, we demonstrated the importance of visual aspects of character design and their impact on persuasion outcomes in Sci-Fi narratives, a huge genre for children and video games in general. More generally, our results contribute to media effects theories by expanding our understanding of the conditions under which audiences will engage with characters of different appearances. Furthermore, we believe that the parallel work could and should be done with live action media. On a psychological level, positive racial representation of just out-group children characters may result in negative reactions among children of color and may even hinder the health behaviors presented in the media. Therefore, for effective public health communication, creative racial diversification is needed. Fantastical characters that take full advantage of the affordances of animated media can lead to greater narrative engagement and wishful identification. When media creators design Sci-Fi characters, ambiguous-looking characters with a fantastical color palette may resonate more with predominantly Black and Latino children when compared to racially unambiguous design featuring White children characters.

## Supplementary Material

jqad030_Supplementary_Data

## Data Availability

The data that support the findings of this study are available from the corresponding author, A.S.L., upon reasonable request.
