# Supplementary material for: The effect of animated Sci-Fi characters’ racial presentation on narrative engagement, wishful identification, and physical activity intention among children
Source: J Commun. 2023 Oct 25;74(2):160–72. doi: 10.1093/joc/jqad030 (PMC11001265; doi:10.1093/joc/jqad030)
Supplement: jqad030_Supplementary_Data [file jqad030_supplementary_data.zip › Supplementary Table 1.docx]

Supplementary Table 1: Questionnaire Measures (R: Reverse Coded)

*Narrative Engagement*:

1. It was easy for me to understand what happened in the story.
2. I understood the characters.
3. I understood the story.
4. R: I found my mind wandering while the story was on.
5. R: While the story was on, I found myself thinking about other things.
6. R: I had a hard time keeping my mind on the story.
7. During the story, my body was in the room, but my mind was in the story world.
8. The story created a new world that suddenly disappeared when the story ended.
9. Sometimes the story world was closer to me than the real world.
10. The story influenced me emotionally.
11. When a main character in the story succeeded, I felt happy.
12. When a main character in the story suffered in some way, I felt sad.
13. I felt sorry for some of the characters in the story

*Wishful Identification*:

1. I'd like to do the kinds of things he/she does in the story.
2. He/she is the sort of person I want to be like.
3. I wish I could be more like him/her.

*Physical Activity Intention*: If there is an active video game with this story as its plot, what would you do?

1. I intend to exercise through this active video game.
2. I plan to exercise through this active video game.
3. I would like to play this active video game.
4. I intend to play this active video game.
5. I would try to play this active video game.

*Social Desirability*:

1. I like everyone I know.
2. I am always kind.
3. I always have good manners.
4. I am always good.
5. I am always nice to everyone.
6. I tell the truth every single time.
7. I never get angry.
8. I never say things I shouldn’t.
9. I never lie.

*Multigroup Ethnic Identity Measures*:

1. I have spent time trying to find out more about my ethnic group’s history, traditions, and customs.
2. The organizations or social groups in which I am active include mostly members of my own ethnic group.
3. I have a clear sense of my ethnic background and what it means for me.
4. I think a lot about how my life will be affected by my ethnic background.
5. I am happy that I am a member of my ethnic group.
6. I have a strong sense of belonging to my own ethnic group.
7. I understand pretty well what my ethnic group membership means to me.
8. I have often talked to other people in order to learn more about my ethnic background.
9. I have a lot of pride in my ethnic group.
10. I frequently enjoy special food, music and customs of my ethnic group.
11. I feel a strong attachment towards my own ethnic group.
12. I feel good about my ethnic background.
13. I have often done things that will help me understand my ethnic background better.

Readability Statistics:

*Words per Sentence*: 10.1

*Characters per Word*: 4.0

*Flesch Reading Ease*: 81.3

*Flesch-Kincaid Grade Level*: 4.4

*Passive Sentences*: 2.3%
