# Supplementary figures and images for: The effect of animated Sci-Fi characters’ racial presentation on narrative engagement, wishful identification, and physical activity intention among children
Source: J Commun. 2023 Oct 25;74(2):160–72. doi: 10.1093/joc/jqad030 (PMC11001265; doi:10.1093/joc/jqad030)

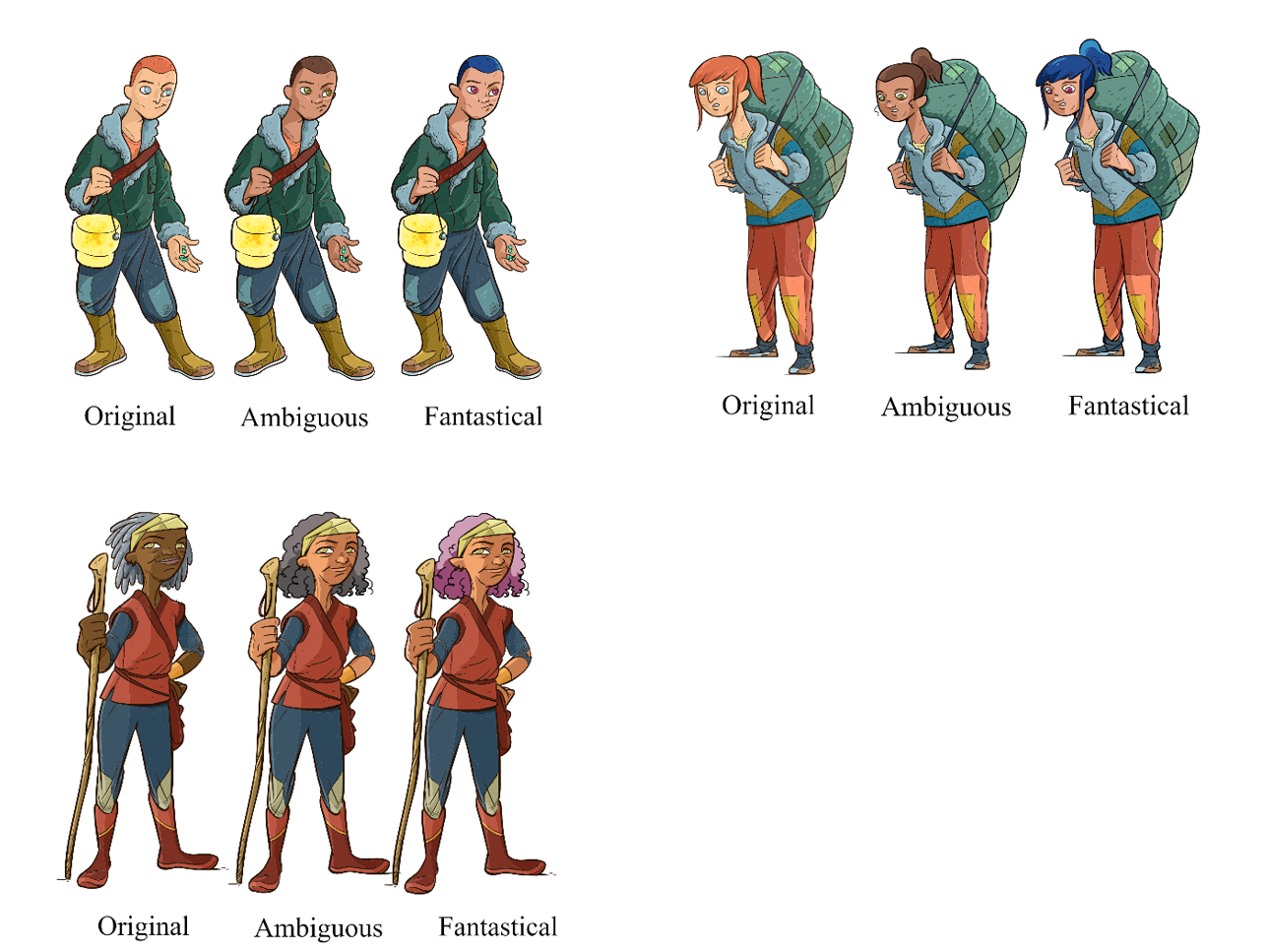

Supplement: jqad030_Supplementary_Data [file jqad030_supplementary_data.zip › Supplementary Figure 1.png]

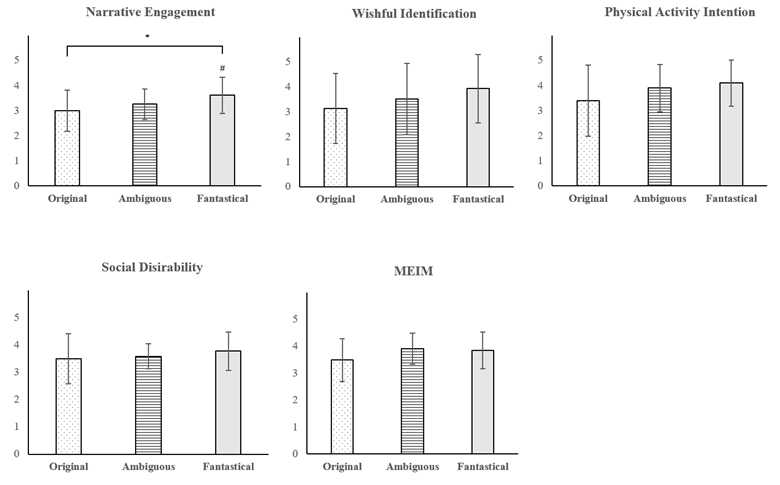

Supplement: jqad030_Supplementary_Data [file jqad030_supplementary_data.zip › Supplementary Figure 2.png]

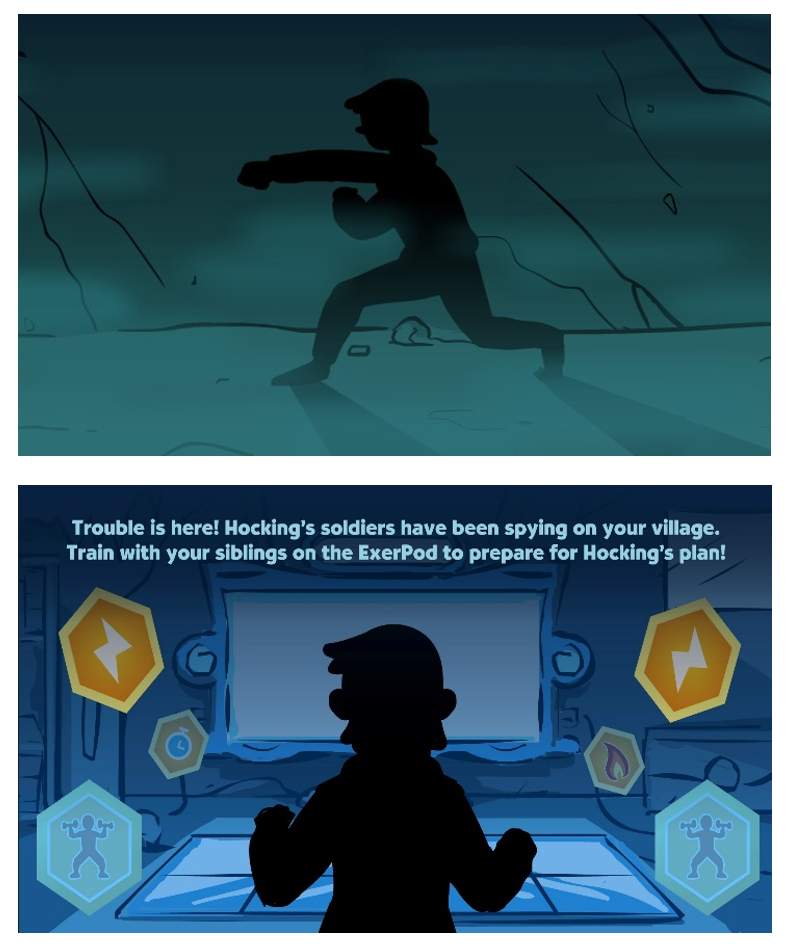

Supplement: jqad030_Supplementary_Data [file jqad030_supplementary_data.zip › Supplementary Figure 3.png]
